# Genome-wide identification, characterization and transcriptional profile of the *SWEET* gene family in *Dendrobium officinale*

**DOI:** 10.1186/s12864-023-09419-w

**Published:** 2023-07-06

**Authors:** Li Hao, Xin Shi, Shunwang Qin, Jiahong Dong, Huan Shi, Yuehua Wang, Yi Zhang

**Affiliations:** 1 College of Food and Biological Engineering, Chengdu University, Chengdu, 610106 PR China; 2 China-Croatia ‘Belt and Road’ Joint Laboratory on Biodiversity and Ecosystem Services, Chengdu Institute of Biology, Chinese Academy of Sciences, Chengdu, 610041 PR China

**Keywords:** Sugar transporter, Polysaccharides, Stem, Expression pattern, Biotic and abiotic stress

## Abstract

**Background:**

*Dendrobium officinale* Kimura et Migo (*D. officinale*) is a well-known traditional Chinese medicine with high content polysaccharides in stems. The SWEET (Sugars Will Eventually be Exported Transporters) family is a novel class of sugar transporters mediating sugar translocation among adjacent cells of plants. The expression patterns of SWEETs and whether they are associated with stress response in *D. officinale* remains uncovered.

**Results:**

Here, 25 *SWEET* genes were screened out from *D. officinale* genome, most of which typically contained seven transmembrane domains (TMs) and harbored two conserved MtN3/saliva domains. Using multi-omics data and bioinformatic approaches, the evolutionary relationship, conserved motifs, chromosomal location, expression patterns, correlationship and interaction network were further analyzed. *DoSWEETs* were intensively located in nine chromosomes. Phylogenetic analysis revealed that DoSWEETs were divided into four clades, and conserved motif 3 specifically existed in DoSWEETs from clade II. Different tissue-specific expression patterns of *DoSWEET*s suggested the division of their roles in sugar transport. In particular, *DoSWEET5b*, *5c*, and *7d* displayed relatively high expression levels in stems. *DoSWEET2b* and *16* were significantly regulated under cold, drought, and MeJA treatment, which were further verified using RT-qPCR. Correlation analysis and interaction network prediction discovered the internal relationship of *DoSWEET* family.

**Conclusions:**

Taken together, the identification and analysis of the 25 *DoSWEETs* in this study provide basic information for further functional verification in *D. officinale*.

**Supplementary Information:**

The online version contains supplementary material available at 10.1186/s12864-023-09419-w.

## Introduction

*Dendrobium officinale* Kimura et Migo is a widely known traditional Chinese medicine in Orchidaceae [1], whose stems harbor great medicinal and economic value due to its high content of active polysaccharides [2, 3]. The 2-O-acetylglucomannan is the main form of polysaccharides in *D. officinale* stems [4]. Polysaccharides content was reported to gradually accumulate from vegetative growth to mature stage in stems, peaking in the 12th month after sprouting [5, 6]. In higher plants, polysaccharides are synthesized from carbohydrates produced in leaves [7]. Firstly, photosynthetic sucrose is loaded to the phloem by sugar transporters. Then, with the help of hydrolases and synthetases, sucrose is unloaded from the phloem into sink tissues for storage as different types of sugars [8]. Profiting from the development of multi-omics analysis, high quality genome of *D. officinale* have become available [9] and vital insights into the biosynthesis pathway and regulation of bioactive polysaccharides have been achieved [5, 6, 10–14]. However, the molecular mechanisms of how sugar is transported from leaves to stems for polysaccharides accumulation in *D. officinale* remain largely unknown.

Monosaccharide transporters (MSTs), sucrose transporters/sucrose carriers (SUCs/SUTs) [8, 15], and sugars will eventually be exported transporters (SWEETs) are three major types of sugar transporters. *DoHT1*, encoding a MST in *D. officinale*, exhibited the most dominant expression level in leaves [16]. Wang et al. [13] found eight *SUT* genes in *D. officinale* and most of them were expressed in flowers, indicating that *DoSUTs* might mainly function in the development of floral organs. The *SWEET* family is a new group of sugar transporters facilitating the translocation of sugars across the intracellular or plasma membranes in plants and animals [17, 18]. Different from SUTs and MSTs, SWEETs can transport not only sucrose but also monosaccharides in bi-direction [8]. The plant SWEET proteins typically contain seven TMs and harbor two conserved MtN3/saliva domain (PF03083), which are linked by the 4th TM [19]. SWEET proteins are phylogenetically divided into four clades (Clade I-IV) [17]. A number of studies have shown that SWEETs participate in various physiological activities through mediating long-distance transport of sugar [20], such as seed filling [21], fruit development [22], nectar secretion [23], pollen nutrition [24], response to abiotic and biotic stresses [25–28]. In *Arabidopsis thaliana* (L.) Heynh., SWEETs belonging to different clades appear selective preferences for sugar types. SWEET proteins from Clade I and II are involved in hexose translocation, Clade III SWEETs specifically transport sucrose, and Clade IV SWEETs tend to transport fructose [8]. Wang et al. [29] identified 22 SWEET family members in *D. officinale* genome, however, no further researches have been done to uncover more details about their structural characteristics, evolutionary relationship, expression profiles, and biological functions.

Although we have had the basic understanding of polysaccharide synthesis, little is known about the mechanisms underlying sugar transport and polysaccharide accumulation in *D. officinale*. In this work, based on the chromosome-scale genome of *D. officinale* [9], the *SWEET* gene family was screened and analyzed using multiple bioinformatic tools and online websites. Our findings do favor to further functional analysis and application of *D. officinale* SWEET family in molecular marker-assisted selection for breeding new varieties with high polysaccharides content and stress resistance.

## Results

### Identification and phylogenetic analysis of ***SWEET*** genes in ***D. officinale***

To identify the *SWEET* gene family members in *D. officinale*, the conserved MtN3/saliva domain (PF03083.hmm) was used to align with the public genome database (accession number GCA_019514585.1) [9] downloaded from NCBI. In total, 25 *SWEET* family members were screened from the chromosome-level genome of *D. officinale* by bioinformatic analysis and were renamed based on the homology with AtSWEETs. The detailed basic physicochemical properties information of DoSWEETs were discovered and listed in Additional file 1, including gene name, chromosomal location, coding sequence length, and protein characteristics (size, molecular weight, isoelectric point, sub-cellular localization, number of transmembrane domains and MtN3/saliva domain). The *DoSWEETs* were distributed on nine chromosomes, and chromosome 11 contained the largest number of members (six *DoSWEETs*). The protein lengths of DoSWEETs ranged from 139 to 297 amino acids and the predicated molecular weights (Mw) were 15.83–33.11 KDa. The isoelectric points (pI) of DoSWEETs were all larger than 8.00, indicating that they were neutral or basic proteins (Additional file 1). Multiple online websites were used to predict the sub-cellular localization of DoSWEETs. Most DoSWEETs were located in cell membrane and contained seven transmembrane domains and two MtN3/saliva domains.

**Fig. 1:**
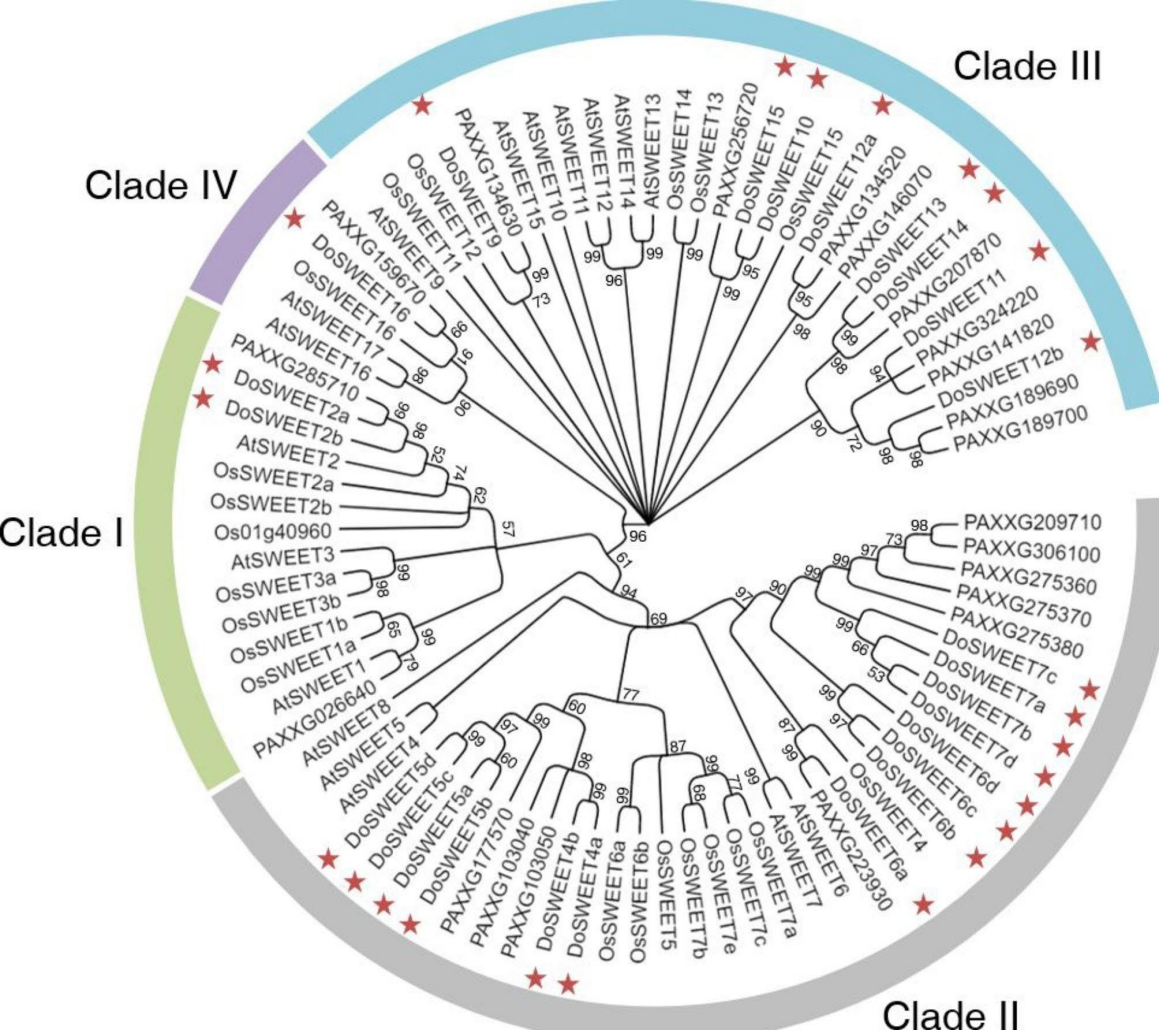
Phylogenetic analysis of SWEETs proteins from *D. officinale*, *A. thaliana*, *O. sativa* and *P. equestris*. The un-rooted phylogenetic tree was constructed using Maximum Likelihood Estimate method in MEGA 11. The bootstrap value was set as 1000 replicates. Percentages of replicate trees in which the associated sequences clustered together in the bootstrap test are shown next to the branches. Clade I, II, III, and IV are marked by green, gray, blue, and purple, respectively. DoSWEETs are marked in red stars. The abbreviations of species names are as follows: Do, *Dendrobium officinale*; At, *Arabidopsis thaliana*; Os, *Oryza sativa*; the proteins started with PA are PeSWEETs from *Phalaenopsis equestris*

**Fig. 2:**
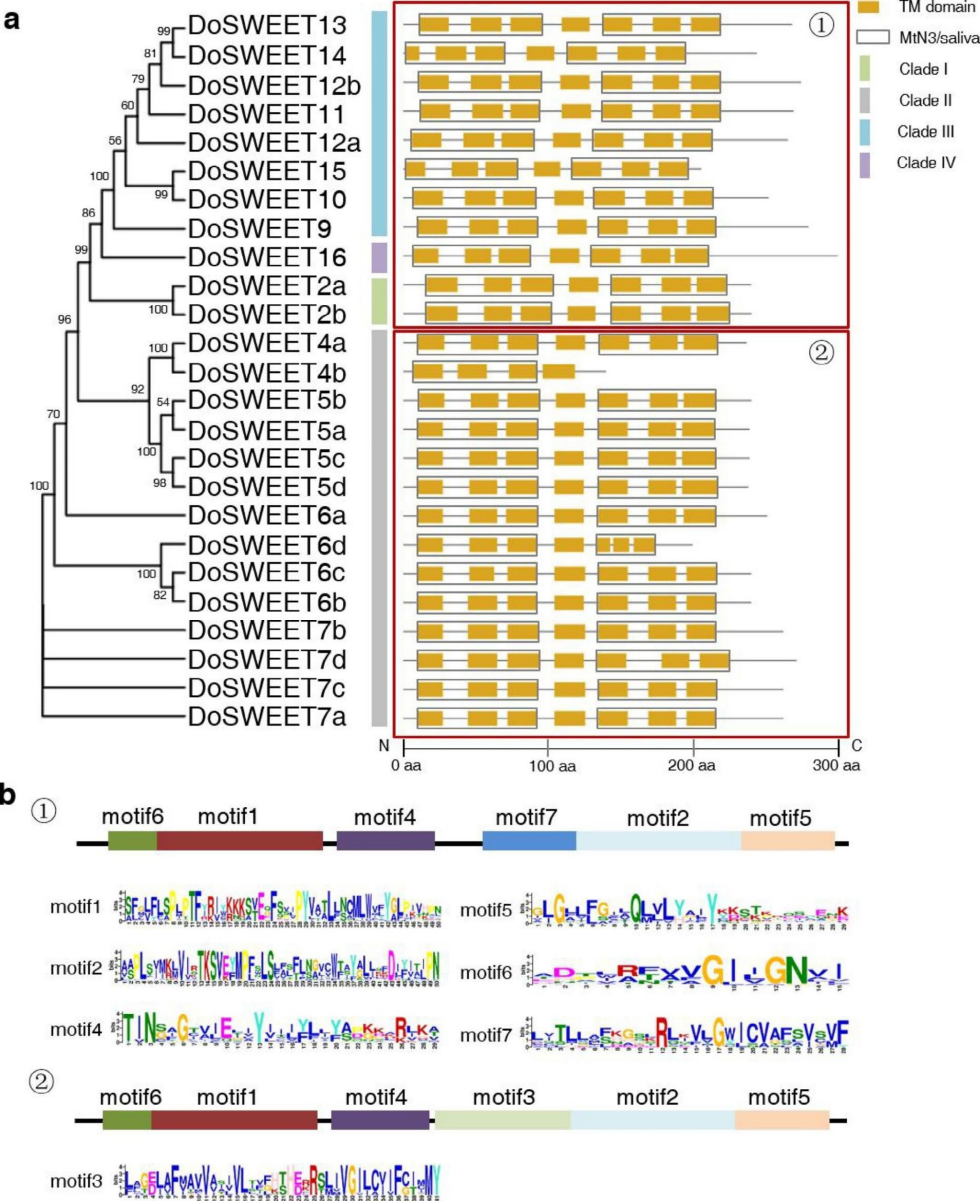
Phylogenetic relationship and conserved domain analysis of DoSWEETs. **a** Phylogenetic relationship and conserved domain distribution of DoSWEETs. An un-rooted phylogenetic tree was constructed using Maximum Likelihood Estimate method with a bootstrap analysis of 1000 replicates in MEGA 11, based on the full-length amino acid sequences of DoSWEETs. Percentages of replicate trees in which the associated sequences clustered together in the bootstrap test are shown next to the branches. Clade I, II, III, and IV are marked by green, gray, blue, and purple, respectively. TM domains are highlighted in orange boxes and MtN3/saliva domains are marked with gray frames. TM, transmembrane domain. **b** The conserved motifs identified by MEME tools in DoSWEETs. Motif 1, 2, 4, 5, 6, 7 were commonly presented in DoSWEETs marked with red frame ① and motif 1, 2, 3, 4, 5, 6 were commonly presented in DoSWEETs marked with red frame ②. The length of each box is proportional to the size of the motif. The sequence logos of motif 1–7 are shown

**Fig. 3:**
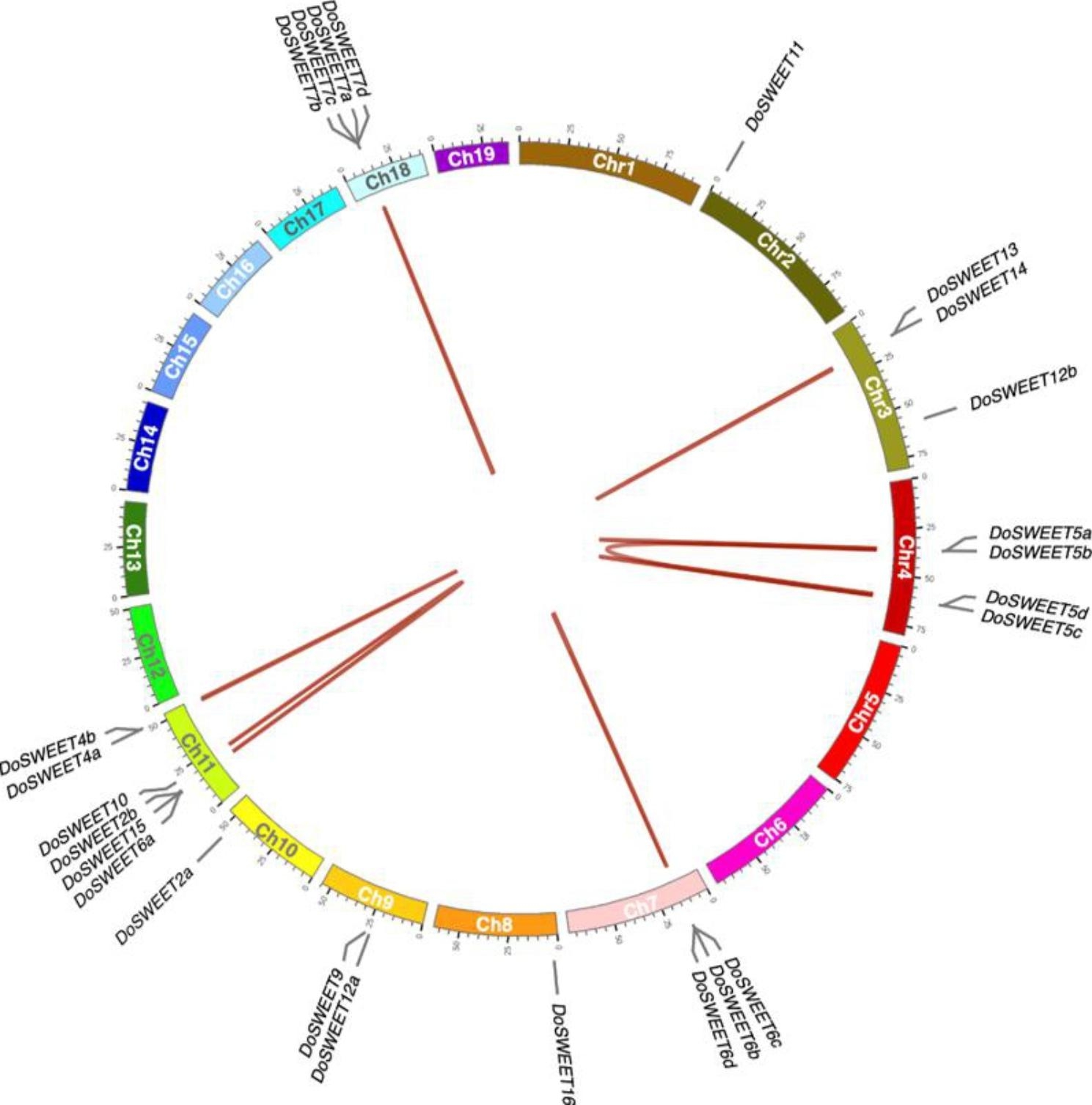
Localization and synteny of *DoSWEETs* in *D. officinale* genome. Different chromosomes are represented by boxes with different colors. The gray solid lines at the edge of the boxes indicate the location of the genes. The duplicated gene pairs, whose alignable sequence covered over 75% of the longer gene, and at the same time, the similarity of aligned regions was more than 75%, are linked with the red lines inside the circle

To investigate the evolutionary relationship of DoSWEETs, an un-rooted maximum likehood tree was constructed using the full length of 84 SWEET proteins from *A. thaliana* (dicot), *Oryza sativa* L. (monocot), *Phalaenopsis equestris* (Schauer) Rchb.f. (the model plant of orchidaceae species), and *D. officinale*. All SWEETs were classified into four clades. In all clades, DoSWEETs formed subclade with SWEETs from *P. equestris*, indicating the closer relationship (Fig. 1). Clade II was the the largest clade and contained 14 DoSWEETs (DoSWEET4-7) with four pairs of paralogs (DoSWEET4a-DoSWEET4b, DoSWEET5a-DoSWEET5b, DoSWEET5c-DoSWEET5d, and DoSWEET6b-DoSWEET6c), followed by clade III with eight members (Fig. 2a). The DoSWEETs (DoSWEET9-15) in clade III comprised two pairs of paralogs (DoSWEET10-DoSWEET15, and DoSWEET13-DoSWEET14). Clade I had 2 DoSWEETs (DoSWEET2a and 2b), which was also identified as a pair of paralogs (Fig. 2a). Clade IV was the smallest one, containing one DoSWEET (DoSWEET16) (Figs. 1 and 2a). The phylogenetic tree with actual branch lengths and scale was shown in Additional file 2 (Fig. S1). Except for DoSWEET2a-DoSWEET2b, the members of other paralog pairs were located on the same chromosomes, respectively (Additional file 1 and Fig. 3). To better study the evolution of *DoSWEET* gene family, gene duplication events were further explored (Fig. 3 and Additional file 3). Based on the close distance and high similarity, ten gene pairs (*DoSWEET5a*-*DoSWEET5b*, *DoSWEET5c*-*DoSWEET5d*, *DoSWEET6b*-*DoSWEET6c*, *DoSWEET6c*-*DoSWEET6d*, *DoSWEET7a*-*DoSWEET7b*, *DoSWEET7a*-*DoSWEET7c*, *DoSWEET7b*-*DoSWEET7c*, *DoSWEET7b*-*DoSWEET7d*, *DoSWEET7c*-*DoSWEET7d*, and *DoSWEET13*-*DoSWEET14*) were considered to be evolved from tandem duplication events (Fig. 3 and Additional file 1, 3). And the other seven duplicated gene pairs were more likely to expand through segmental duplications due to their longer physical distance (larger than 100 kb) with each other (Fig. 3 and Additional file 1, 3). To investigate the selective evolutionary pressure on *DoSWEET* gene divergence after duplication, the non-synonymous and synonymous substitutions per site (Ka and Ks values), and Ka/Ks ratio were calculated (Additional file 3). We found that Ka/Ks ratios of all those gene pairs were less than 1, indicating that the occurrence rate of synonymous substitutions was higher than that of non-synonymous substitutions. This results revealed that the purifying selection had the primary influence on *DoSWEET* gene family in the evolution.

### Conserved domain and motif analysis of ***DoSWEETs***

All of the DoSWEETs harbor seven TMs and contain two MtN3/saliva domains, except for DoSWEET4b (Additional file 1 and Fig. 2a). Multiple alignment analysis by DNAMAN software showed that the sequences of seven TMs were conserved among DoSWEETs (Additional file 2: Fig. S2). To further analyze the conserved TMs, hydrophobicity and hydrophilicity values were estimated and the results indicated that the TM regions were more hydrophobic (Additional file 2: Fig. S3).

Ten conserved motifs were predicted by MEME (Additional file 2: Fig. S4). Motif 1, 2, 4, 5, 6 were shared in all DoSWEETs. Motif 7 existed in DoSWEETs from clade I, III and IV, while motif 3 was unique in clade II (Fig. 2), which might be related to functional divergence between clade II and the other clades. The distribution and sequence logo of seven conserved motifs are shown in Fig. 2b.

### Tissue-specific expression pattern of ***DoSWEETs***

**Fig. 4:**
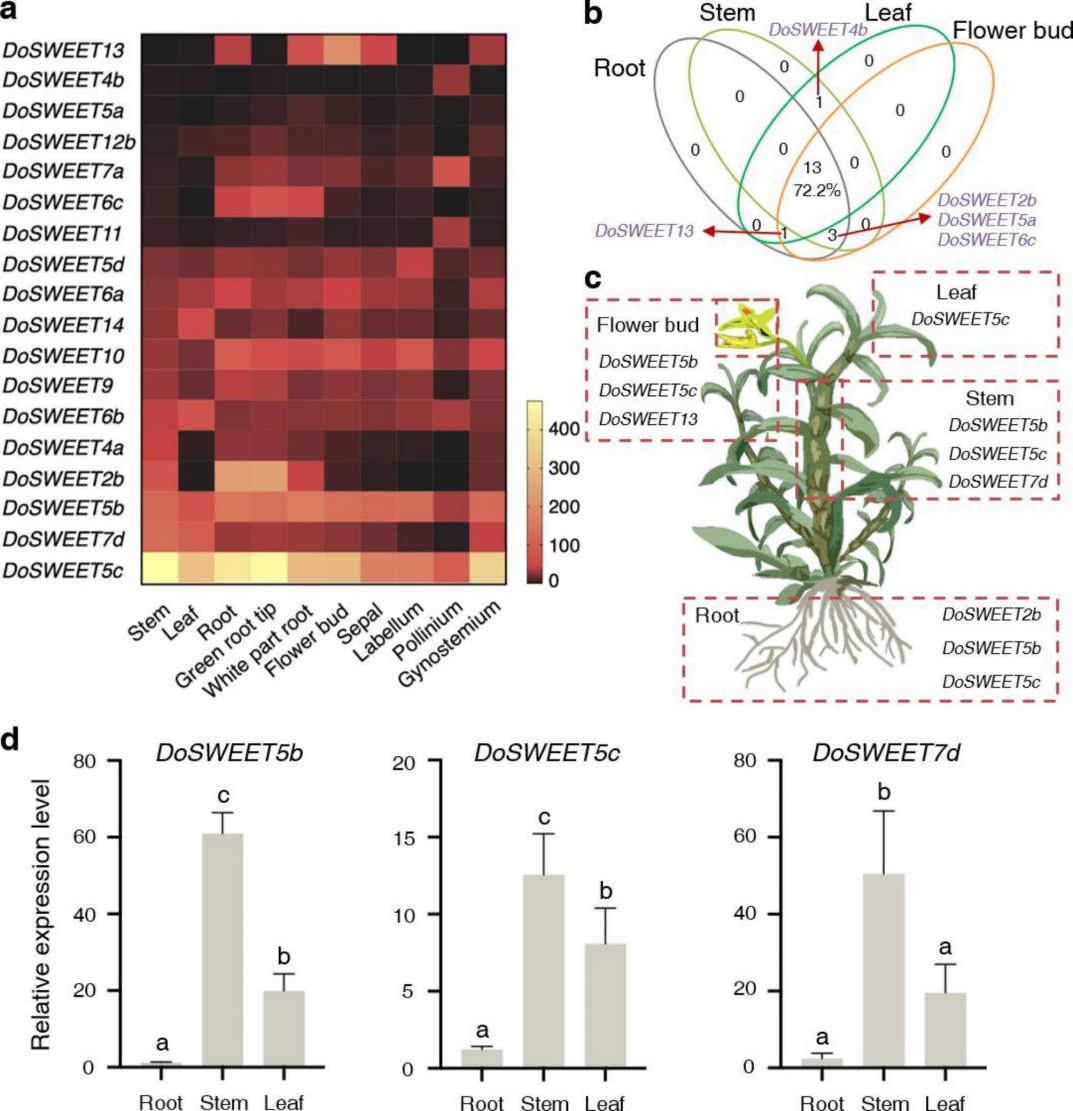
Expression patterns of *DoSWEETs* in different tissues. **a** A heatmap displaying the FPKM values of *DoSWEETs* in different tissues of *D. officinale*. One biological replicate per sample. **b** A venn diagram showing the expression of *DoSWEETs* in the roots, stems, leaves, and flower buds. The majority of *DoSWEETs* (72.2%) were expressed in the four tissues analyzed. FPKM values greater than 1 were counted. **c** The highly expressed *DoSWEETs* (FPKM > 100) in the roots, stems, leaves, and flower buds. **d** Expression detection of *DoSWEET5b*, *DoSWEET5c*, and *DoSWEET7d* in the roots, stems, and leaves by RT-qPCR. Bars represent means ± SD (n = 3). Different letters above each column indicate the significant differences based on Duncan’s test (*P* < 0.05)

To investigate the tissue-specific expression patterns of *DoSWEETs*, the FPKM values were downloaded from OrchidBase 4.0 [30] and were visualized in the heatmap (Fig. 4a and Additional file 4). Eighteen *DoSWEETs* were detected in at least one of the ten tissues. All of the detected *DoSWEETs* displayed tissue-specific expression patterns (Fig. 4a). *DoSWEET5c* was the most abundant one in all of the ten tissues (Fig. 4a and Additional file 4). The most of *DoSWEETs* (72.2%) were expressed in roots, stems, leaves and flower buds from *D. officinale* (Fig. 4b). *DoSWEET4b* and *11* were highly expressed in the pollinia, whereas *DoSWEET6a*, *7d*, *9*, and *14* showed the opposite expression patterns, with the lowest level in the pollinia (Fig. 4a). Stem, rich in polysaccharides, is the medicinal part of *D. officinale*. *DoSWEET5b*, *DoSWEET5c*, and *DoSWEET7d* displayed relatively high expression levels in the stems (FPKM > 100) (Fig. 4c), which were determined using RT-qPCR (Fig. 4d), indicating their potential roles in polysaccharide accumulation in this tissue. In addition, *DoSWEET2b*, *DoSWEET5b*, and *DoSWEET5c* were highly expressed in the roots and green root tips (FPKM > 100). Especially *DoSWEET2b*, the expression level was dominant in the roots compared to other tissues (Fig. 4c and Additional file 4).

### ***DoSWEET2b*** and ***DoSWEET16*** were regulated by low temperature, drought stress and MeJA treatment

**Fig. 5:**
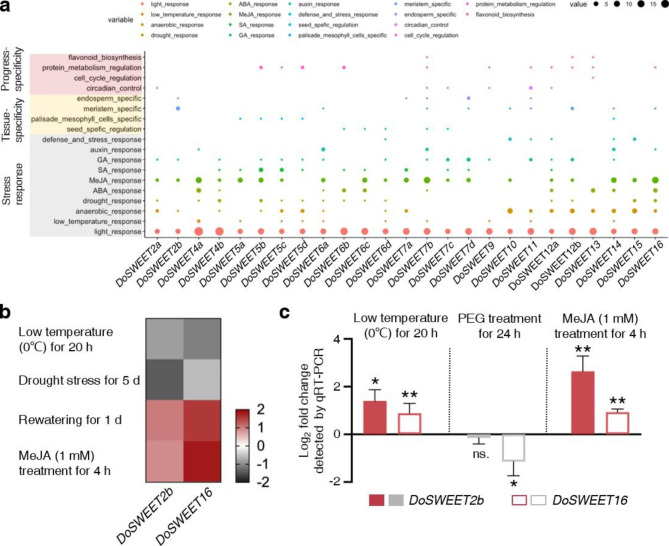
Stress-responsive analysis of *DoSWEETs*. **a*** Cis*-acting elements analysis of *DoSWEETs* promoters using PlantCARE. The 2000 bp upstream sequences from the translational initiation codon of *DoSWEETs* were analyzed. The identified elements were divided into three classes, including stress-response, tissue-specificity, and progress-specificity. Dot color and size indicate the type and number of the elements, respectively. **b*** DoSWEET2b* and *DoSWEET16* were regulated by low temperature, drought stress, and MeJA treatment. Gray and red represent down- and up-regulation, respectively. The downloaded RNA-seq datasets of low temperature and MeJA treatment contained three replicates, while the datasets of drought stress had only one biological replicate. Log_2_(mean FPKM of experimental group/control group) values were used to draw the heatmap. **c** The log_2_ fold change of *DoSWEETs* expression under different stresses detected by RT-qPCR. PEG treatment was used to simulate the drought stress conditions. Bars represent means ± SD (n = 3). The symbols above each column indicate the significant differences compared with control based on Student’s *t*-test (**P < 0.01; *P < 0.05; ns. represented no significance)

*SWEETs* have been reported to participate in stress responses [31]. In order to identify the *cis*-acting elements on the promoters of *DoSWEETs*, the 2000 bp upstream sequences from the translational initial codon were extracted and analyzed using the online website PlantCARE, (http://bioinformatics.psb.ugent.be/webtools/plantcare/html/). Eighteen types of *cis*-acting elements were identified (Additional file 5) and were further classified into three categories, including stress response, tissue-specificity, and progress-specificity. Each *DoSWEET* harbored at least five types of *cis*-acting elements on its promoter (Fig. 5a).

To further explore the responses of *DoSWEETs* under different stresses, the transcriptome sequencing data under low temperature [32], drought stress [33], cadmium stress [34] and MeJA treatment [35] were obtained from NCBI and the FPKM values were calculated to assess *DoSWEETs* expression levels. Only *DoSWEET2b* and *DoSWEET16* were found to respond to stresses and the change trends were similar with each other (Fig. 5b and Additional file 6). After treatment with 0℃ for 20 h, *DoSWEET2b* and *DoSWEET16* were down-regulated by 0.84 and 1.10 fold, respectively (Fig. 5b and Additional file 6). Drought stress also caused the down-regulation of the two genes, and their expression increased after re-watering (Fig. 5b). When treated with exogenous MeJA, *DoSWEET2b* was induced by 0.90 fold and *DoSWEET16* expression was 1.80-fold higher than the control (Fig. 5b and Additional file 6). The result is consistent with the MeJA-responsive elements predicted on their promoters (Fig. 5a). Furthermore, the responsive intensity was positively related to the number of MeJA-responsive elements (Fig. 5a and b). No *DoSWEETs* were found to respond to cadmium stress. To verify the responses of *DoSWEET2b* and *DoSWEET16* under the above stresses, RT-qPCR analysis was conducted. The results showed that the expression changes of *DoSWEET2b* and *DoSWEET16* under MeJA treatments were consistent with that in transcriptome data, while the expression under cold stress exhibited the opposite trend (Fig. 5b and c). PEG treatment was used to simulate the drought stress conditions. RT-qPCR confirmed the decrease of *DoSWEET16* expression after PEG treatment. However, no significant change of was detected for *DoSWEET2b* (Fig. 5b and c).

### Potential SWEET dimers formed when functioning

**Fig. 6:**
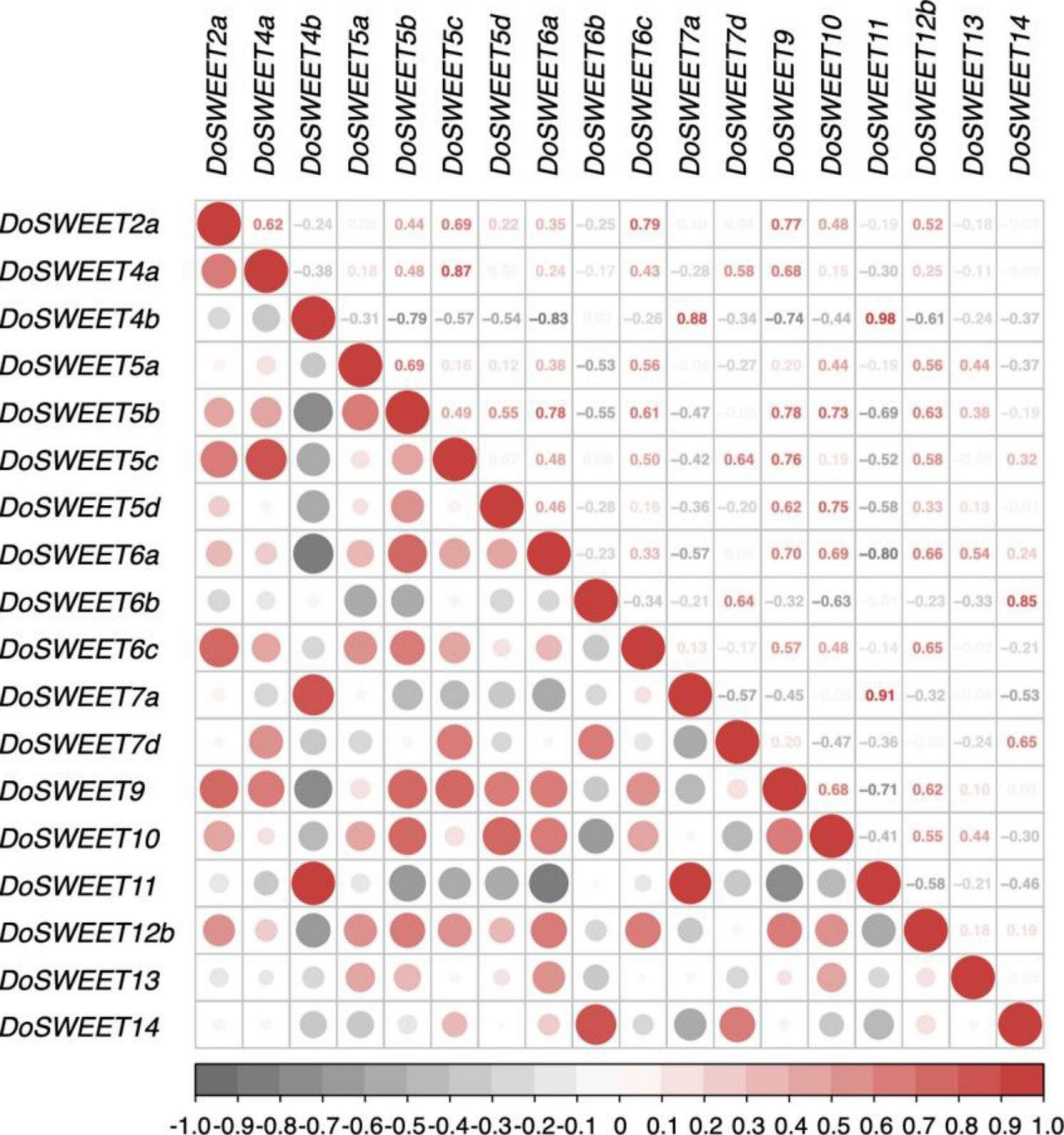
Correlation analysis of *DoSWEETs* using *corrplot* R package. The co-expression relationship of *DoSWEETs* were analyzed using *corrplot* R package based on the FPKM values in different tissues and under different stresses. The correlation coefficients are shown in the upper right portion of the diagram and visualized in the bottom left portion. Bigger dot size represents higher correlation coefficient. Red and gray dots represent the positive and negative correlationship, respectively

**Fig. 7:**
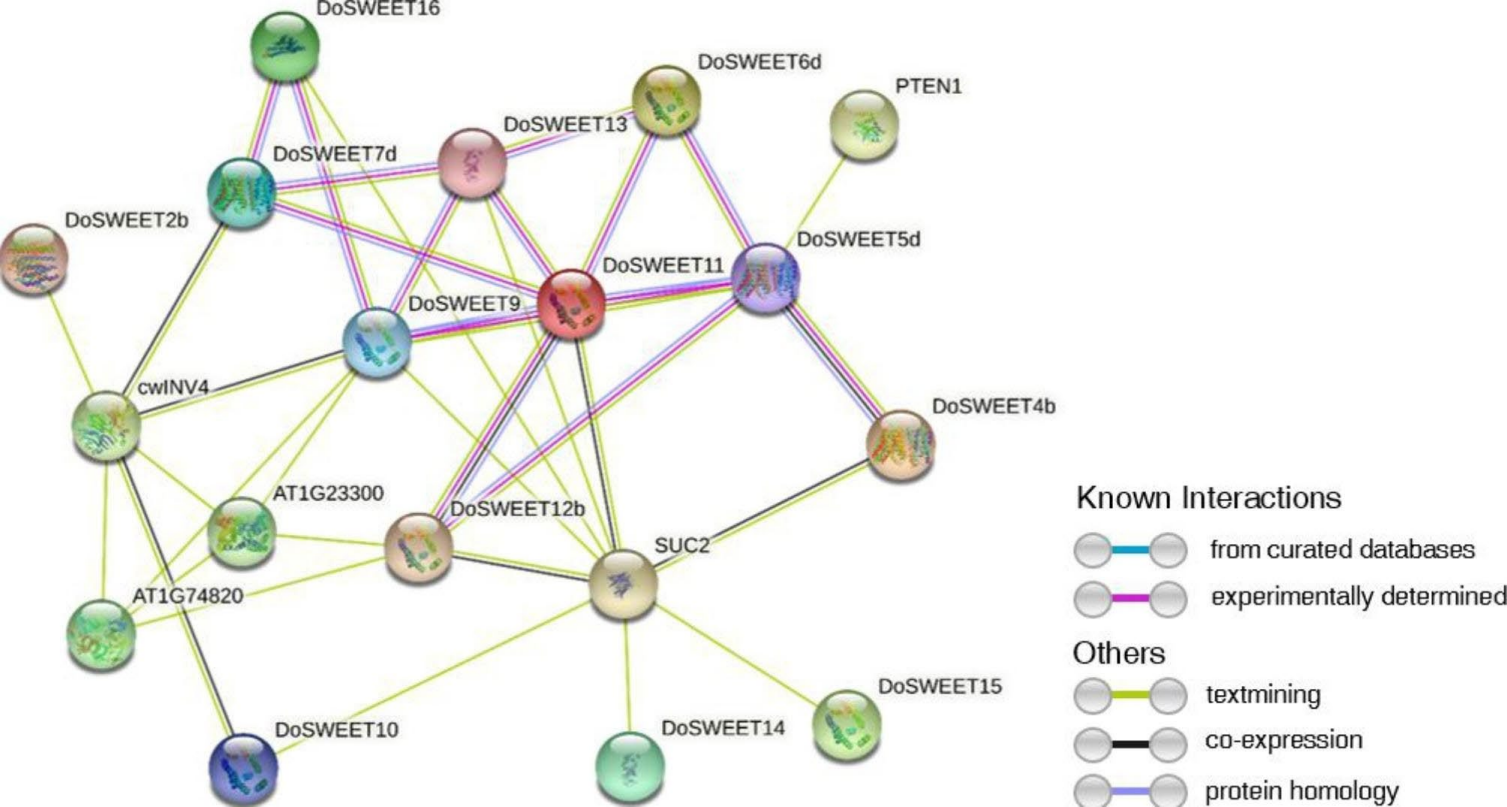
Interaction network prediction of *DoSWEETs*using STRING. The interactions were predicted based on the homologous genes of *DoSWEETs* in *A. thaliana*. The lines with different colors represent different types of interaction evidence

Function of SWEET sugar transporter requires a pore consisting of at least two SWEETs [36]. To find out the potential SWEET dimers in *D. officinale*, the correlation analysis and interaction network prediction were performed. Correlation analysis indicated that *DoSWEET4a*-*DoSWEET5c*, *DoSWEET4b*-*DoSWEET7a*, *DoSWEET4b*-*DoSWEET11*, *DoSWEET6b*-*DoSWEET14*, and *DoSWEET7a*-*DoSWEET11* were co-expressed with highly positive correlation coefficients of 0.87, 0.88, 0.98, 0.85, and 0.91, respectively. By contrast, *DoSWEET4b*-*DoSWEET6a* and *DoSWEET6a*-*DoSWEET11* showed negative correlations with correlation coefficients of -0.83 and − 0.80, respectively (Fig. 6). To analyze the functional and physical interaction of DoSWEETs, STRING software was used to draw the interaction network map. Multiple interrelationships were found among DoSWEETs. For example, more than three types of interaction evidence were identified between DoSWEET11 and other DoSWEETs, including DoSWEET5d, DoSWEET6d, DoSWEET7d, DoSWEET9, DoSWEET12b, and DoSWEET13 (Fig. 7). All those DoSWEET pairs were potential to form dimers when functioning. In addition, DoSWEET4b, DoSWEET9, DoSWEET10, DoSWEET11, DoSWEET12b, DoSWEET13, DoSWEET14, DoSWEET15, and DoSWEET16 were related to SUC2, a sucrose transport protein in *A. thaliana* [15] (Fig. 7), indicating their potential function in sugar translocation.

## Discussion

As an important energy source for plant growth and development, sugar is synthesized in the leaves and transported into non-photosynthetic sink tissues via symplastic pathway and apoplastic pathway [37]. Over the past 30 years, the molecular mechanisms of sugar apoplastic transport have been uncovered in model plants [17, 38]. Polysaccharide is one of the most valuable medicinal ingredients in *D. officinale* stems, it is meaningful to screen candidate *SWEET* genes in *D. officinale* for clarification of their roles in polysaccharide accumulation.

In 2010, Chen et al. [17] first identified SWEET1 as a membrane-located glucose uniporter in *A. thaliana*, mediating glucose efflux across the membrane. From then on, benefiting from sequencing technologies and bioinformatic tools, the SWEET family have been widely identified in many other plants [18]. In this study, 25 *SWEET* members were found in *D. officinale* genome (Additional file 1). Similarly, other species in monocots generally have more than 20 members in the SWEET family. For example, there are 21 members in *O. sativa* [39], 24 in *Zea mays* (L.) [21], 23 in *Sorghum bicolor* (L.) Moench [40], 25 in *Musa acuminata* Colla [41], 39 in *Ananas comosus* (L.) Merr. [22], and 22 in *Saccharum spontaneum* (L.) [42]. Specially, in *P. equestris*, another orchid species, only 16 putative *SWEET* genes were discovered [29]. Compared to clade I, III and IV, DoSWEETs from clade II specifically harbored the conserved motif 3 and showed obvious gene expansion (Figs. 2 and 3), which is similar to the *SWEET* family in *Dendrobium chrysotoxum* Lindl. [43]. In *A. thaliana*, members of this clade (SWEET4-8) tend to transport monosaccharides [8]. These findings suggest that members of DoSWEET4-7 subfamily may be related to monosaccharides translocation and abundant polysaccharides content in *D. officinale* stems. In addition, the conserved motif 3 is likely to be involved in functional divergence during evolution. Duplication is one of the primary driving forces to facilitate the gene expansion and genome evolution [44, 45]. Here, more pairs of tandem duplication *DoSWEETs* were identified than segmental duplication ones, Therefore, tandem duplication is considered to be the main reason for the expansion of the *SWEET* family in *D. officinale*, resulting in the high content of polysaccharides in the stems.

The whole process of sugar transport in plants involves the cooperation of SUTs, MSTs, and SWEETs [31]. The functions and interactions of these sugar transporters are relatively well studied in *A. thaliana*. Therefore, the interaction network of DoSWEETs was predicted based on their homologous SWEETs in *A. thaliana*. Notably, seven of the eight DoSWEETs from clade III were found to interact with SUC2 by textmining and co-expression (Fig. 7). SUT/SUC2 has been identified as a H^+^-sucrose symporter mediating phloem apoplast loading in a previous study [15]. In addition, SWEETs belonging to clade III have been reported to mediate sucrose translocation in *A. thaliana* [8]. In that case, clade III DoSWEETs are most likely to be involved in sucrose loading in the phloem. Apart from DoSWEETs from clade III, DoSWEET4b was also predicted to interact with SUC2 (Fig. 7). Considering the mechanism of SWEETs on sugar transport and the specificity of DoSWEET4b in conserved domain (only harbored one MtN3/saliva domain), we assume that DoSWEET4b may form oligomers with other DoSWEETs when functioning. Correlation analysis showed that *DoSWEET4b* had extremely positive correlation (value = 0.98) with *DoSWEET11* (Fig. 6) which was also predicted to be co-expressed with *SUC2* (Fig. 7). In *A. thaliana*, sucrose is synthesized in the leaves and transported to the apoplast by AtSWEET11 and AtSWEET12, and then is loaded into the phloem for long-distance transport by SUT1/SUC2 [20]. *OsSWEET11* in rice is also associated with sucrose transport [20]. Taken together, we conjecture that DoSWEET4b and DoSWEET11 may form oligomers to participate in sucrose transport in source tissues, which remains to be confirmed by experiments.

The different expression patterns of SWEET members indicate the differentiation of their roles in plant growth and development. *DoSWEET5c* was constitutively expressed in *D. officinale*, with extremely high level in the stems (Fig. 4a and Additional file 4). In model plants, function of stem-expressed *SWEETs* is hardly characterized due to the low sucrose content in their stems, except for *S. bicolor*. To know more about the potential role of *DoSWEET5c*, we screened homologous genes of *DoSWEET5c* in *S. bicolor* genome using the online website Phytozome ver.13 (https://phytozome-next.jgi.doe.gov/blast-search). Five orthologs (*SbSWEET3-6*, *SbSWEET4-1*, *SbSWEET4-3*, *SbSWEET9-3*, and *SbSWEET4-2*) in *S. bicolor* were found with high bitscores (Additional file 7). *SbSWEET4-3* was uniquely expressed in *S. bicolor* stems and was potentially responsible for sucrose unloading from the phloem into the stem apoplast [40]. It has been reported that orthologs with high sequence conservation appear to be functionally similar to each other [46]. So, it is speculated that *DoSWEET5c* might also be involved in sucrose phloem unloading in *D. officinale* stems. In addition, *DoSWEET5b*, the paralogous gene of *DoSWEET5c*, exhibited the similar expression pattern with *DoSWEET5c* (Fig. 4a and Additional file 4) and was highly homologous with *SbSWEET4-3* (Additional file 7). Therefore, we consider that *DoSWEET5b* and *DoSWEET5c* are important for sugar transport and function redundantly in sucrose unloading and polysaccharide accumulation in *D. officinale* stems. *DoSWEET13* was expressed relatively high in the flower buds (Fig. 4 and Additional file 4) and was classified into the same clade with *OsSWEET11*. *OsSWEET11* is reported to be essential for reproductive development of rice [47, 48]. Thus, it is likely that *DoSWEET13* might play a role in the differentiation and development of *D. officinale* flower buds. These findings help to narrow down the range of candidate genes for sugar transport in *D. officinale*. In addition, seven *DoSWEETs* were not detected in the ten examined tissues. There are three explanations for this finding. Firstly, these *DoSWEETs* are likely to express in other specific tissues or development stages. *DoSLR1-1* was only expressed in the seed [49]. *SbSWEET2-1* and *SbSWEET7-1* were specifically expressed in the panicle from the start of heading to 36 days afterward [40]. Secondly, the seven *DoSWEETs* may be stress-responsive genes. For example, *DcSUVH2a* was only detected under high temperature [50]. Thirdly, these unexpressed *DoSWEETs* may have lost their functions during evolution [49]. However, more direct evidences should be provided by subsequent experiments to illustrate the specific function of *DoSWEETs*.

*D. officinale* is an epiphytic orchid and grows in the barks, the branches or the crack in the rocks. Its growth and development usually suffer from biotic and abiotic stresses. Sugar is not only the main energy source necessary for life processes, but also takes part in the responses to diverse stresses. In this study, we mainly focused on the cold, drought, and MeJA-responsive *DoSWEET* genes. *Cis*-acting elements prediction also help us to understand more about *DoSWEET* gene family in stress response. Consistent with the drought-responsive elements found on the promoter, *DoSWEET16* expression decreased under drought stress and then increased after rewatering (Fig. 5b). Although the expression of *DoSWEET2b* also decreased after drought treatment, no relevant elements were predicted on its promoter. Similarly, although no cold-responsible element was found, *DoSWEET2b* and *DoSWEET16* were down-regulated under cold stress. On one hand, the inconsistency may result from the inaccuracy of the prediction. On the other hand, in addition to stresses, gene expression is closely related to many other factors, such as specific tissues and growth stages. Samples of the stress-related RNA-seq data used in this work were all leaves, while the responses of *DoSWEETs* may not be limited to this tissue. To further clarify the roles of *DoSWEETs* in stress response, it is of great significance to detect their expression changes in other tissues of *D. officinale*. *DoSWEET2b*, highly expressed in root (Fig. 4), is orthologous to *AtSWEET2*, *OsSWEET2a*, and *OsSWEET2b*. *AtSWEET2* showed dominant expression level in the tonoplast of roots and was regulated by both pathogen (*Pythium irregulare*) infection and biotic stresses [51]. In addition, symbioses are often formed between orchids and soil mycorrhiza fungi, which is beneficial to plant growth. *DoSWEETs* with high level in the roots may also be related to pathogen nutrition in symbiotic relationship construction. *DoSWEET16*, whose expression decreased in response to cold stress (Fig. 5), is the ortholog of *OsSWEET16*, *AtSWEET17*, and *AtSWEET16* from clade IV (Fig. 1). *AtSWEET16* was located in the vacuole membrane to transport sugar and was down-regulated under cold stress. Plants overexpressing *AtSWEET16* failed to accumulate fructose under cold treatment and showed improved cold tolerance [25]. These results suggest that *AtSWEET16* is involved in strengthening stress resistance through sugar efflux regulation. In this study, although the responses of *DoSWEET2b* and *DoSWEET16* under cold, drought, and MeJA treatment have been confirmed by RT-qPCR, more experiments should be done to uncover the detailed functions and mechanisms.

## Conclusions

In this study, we identified 25 *DoSWEETs* in *D. officinale* and analyzed their characterizations, including evolution relationship, conversed domains, chromosomal localization, and expression patterns, to explore their potential functions. In particular, it was found that *DoSWEET5b*, *5c* and *7d* were enriched in the stems (the site of polysaccharides accumulation). *DoSWEET2b* and *16* responded to different abiotic stresses, which expanded our understandings on the biological functions of *DoSWEETs*. In addition, the potential DoSWEET dimers formed when functioning were predicted. These findings are beneficial to the functional analysis of *DoSWEETs* in the future.

## Materials and methods

### Genome-wide identification of ***SWEET*** gene family in ***D. officinale***

The chromosome-level genome of *D. officinale* was downloaded from NCBI with accession number GCA_019514585.1 (https://www.ncbi.nlm.nih.gov/assembly/GCA_019514585.1, accessed on March 5, 2022) [9]. The hidden Markov model (HMM) of the MtN3/saliva domain (PF03083.hmm) was built from the seed alignment file PF03083.alignment.seed obtained from Pfam database (https://www.ebi.ac.uk/interpro/entry/pfam/PF03083/, accessed on March 5, 2022). And then HMMER 3.1 (https://www.ebi.ac.uk/Tools/hmmer/, accessed on March 6, 2022) was used to screen putative SWEETs in the local pep file of *D. officinale* using PF03083.hmm as a query with a default E-value. All obtained SWEET candidates were submitted to the online website SMART (https://smart.embl.de/, accessed on March 8, 2022) and InterPro (https://www.ebi.ac.uk/interpro/search/sequence/, accessed on March 8, 2022) to confirm the MtN3/saliva domain. The identified *DoSWEETs* were renamed based on the homology with SWEETs from *A. thaliana*.

### Conserved domain and motif analysis of DoSWEETs

For conserved domain analysis, the position information of the transmembrane domain (TM) and MtN3/saliva domain on DoSWEETs was obtained using InterPro (https://www.ebi.ac.uk/interpro/search/sequence/, accessed on March 8, 2022), and then visualized on EvolView (http://www.evolgenius.info/evolview/#/treeview, accessed on March 9, 2022). To identify the conserved motifs, the full length protein sequences of DoSWEETs were analyzed by MEME (https://meme-suite.org/meme/tools/meme) with the following parameters: site distribution being zero or one occurrence per sequence, the number of motifs to find being 10, and the optimum motif width between 6 and 50. All other parameters were set as the default values.

### Physicochemical properties analysis and sub-cellular localization prediction of DoSWEETs

The ExPASy Server (https://web.expasy.org/compute_pi/, accessed on April 25, 2022) [52] was used to compute molecular weight (Mw) and isoelectric point (pI) of DoSWEETs. The Hydrophobicity Profile and Hydrophilicity Profile in DNAMAN 7.0 (accessed on April 23, 2022) were used to compute hydrophobicity and hydrophilicity of DoSWEETs. Plant-mPLoc (http://www.csbio.sjtu.edu.cn/bioinf/plant-multi/, accessed on April 25, 2022) [53], WoLF PSORT (https://wolfpsort.hgc.jp, accessed on March 14, 2023) [54], and ProtComp 9.0 (http://linux1.softberry.com/berry.phtml?topic=protcomppl&group=programs&subgroup=proloc, accessed on March 14, 2023) were employed to predict sub-cellular localization of DoSWEETs.

### Phylogenetic tree construction of DoSWEETs

The amino acid sequences of SWEETs from *A. thaliana* (the model plant in dicots) and *Oryza sativa* L. (the model plant in monocots) were obtained from a previous study [39] and from the online websites (https://www.arabidopsis.org and http://rice.uga.edu, accessed on April 15, 2022). The protein file of *Phalaenopsis equestris* (Schauer) Rchb.f. (another species in orchidaceae) was downloaded from Orchidstra 2.0 (http://orchidstra2.abrc.sinica.edu.tw, accessed on April 18, 2022) [55] and SWEET proteins were screened by HMMER 3.1 (https://www.ebi.ac.uk/Tools/hmmer/, accessed on March 6, 2022) based on PF03083.hmm. Multiple sequence alignment of all SWEET protein sequences, including DoSWEETs, were performed by ClustalW in MEGA 11 (https://www.megasoftware.net, accessed on May 6, 2022) [56]. And then the sequence alignment file was used to construct the un-rooted phylogenetic tree using the Maximum Likelihood Estimate (MLE) method with the Poisson model and a bootstrap analysis of 1000 replicates in MEGA 11. ‘Use all sites’ was chosen for gaps treatment. The sequence alignment file for phylogenetic tree construction is available in Zenodo (10.5281/zenodo.7947133).

### Chromosome localization of ***DoSWEETs*** and Ka/Ks calculation

The sequences of *DoSWEET*s were mapped to *D. officinale* reference genome using ncbi-blast-2.13.0+ (https://www.ftp.ncbi.nlm.nih.gov/blast/executables/blast+/LATEST, accessed on March 15, 2022) to obtain the position information. The duplicated gene pairs of *DoSWEET*s were also identified using ncbi-blast-2.13.0+. Genes matched the following conditions were defined as duplicated gene pairs: a, a length of alignable sequence covered > 75% of the longer gene; b, similarity of aligned regions > 75% [57]. And the duplicated gene pairs with physical distance within 100 kb were considered as tandem duplication gene pairs, otherwise [58]. Then ClustalW (http://www.genome.jp/tools/clustalw/, accessed on March 15, 2022) was employed to align the coding sequences of *DoSWEET* pairs. And the results were used to calculate the non-synonymous substitutions (Ka), synonymous substitutions (Ks) values, and Ka/Ks ratio between each of the gene pairs using KaKs_Calculator 3.0 (accessed on May 12, 2022) with Nei-Gogobori method [59].

The chromosome localization of *DoSWEETs* as well as the tandem duplication gene pairs were visualized using Circos-0.69-6 (accessed on July 12, 2022) [60].

### Tissue-specific expression pattern and stress response analysis of ***DoSWEETs***

The tissue-specific expression data of *DoSWEETs* were downloaded from OrchidBase 4.0 (http://orchidbase.itps.ncku.edu.tw/est/FPKM.aspx?projectname=Dendrobium, accessed on May 23, 2022) [30]. Each tissue only contained one biological replicate in the database. The RNA-seq data of *D. officinale* under low temperature (PRJNA314400) [32], drought stress (PRJNA432825) [33], cadmium stress (PRJNA561268) [34] and MeJA treatment (PRJNA732289) [35] were downloaded from NCBI (https://www.ncbi.nlm.nih.gov, accessed on May 20, 2022). The datasets of low temperature, cadmium stress, and MeJA treatment contained three replicates, while drought stress had only one biological replicate. Data processing and analysis were performed as Hao et al. reported [61]. Briefly, after filtering the adaptor and low-quality reads by trimmomatic 0.39 (accessed on April 15, 2022) [62], the clean data were mapped to *D. officinale* reference genome using HISAT2 2.2.0 (accessed on April 15, 2022) [63]. StringTie 2.2.1 (http://ccb.jhu.edu/software/stringtie/, accessed on April 16, 2022) [64] was employed to calculate fragments per kilobase per million (FPKM) values to assess the gene expression levels. Then, the change of gene expression was calculated as log_2_(mean FPKM of experimental group/control group). The heatmaps in Figs. 4a and 5b were generated from the FPKM values and log_2_(mean FPKM of experimental group/control group) values, respectively, using GraphPad Prism 9 (accessed on March 22, 2022).

Co-expression relationship of *DoSWEETs* was analyzed using *corrplot* R package in RStudio 2022.02.1 + 461 (https://www.rstudio.com, accessed on April 22, 2022) with R 4.1.3 (https://www.r-project.org, accessed on April 22, 2022) based on the FPKM values in different tissues and under different stresses.

### ***Cis***-acting elements prediction on ***DoSWEETs*** promoters

To identify the potential *cis*-acting elements on the promoters, the 2000 bp upstream sequences from the translational initiation codon of *DoSWEETs* were obtained from *D. officinale* genome and subsequently submitted to PlantCARE (http://bioinformatics.psb.ugent.be/webtools/plantcare/html/, accessed on July 6, 2022) [65] to predict the *cis*-regulated elements using the default parameters. The number, distribution, and classification of the elements were further analyzed and visualized using *ggplot2* package in RStudio 2022.02.1 + 461 (https://www.rstudio.com, accessed on April 22, 2022) with R 4.1.3 (https://www.r-project.org, accessed on April 22, 2022).

### Interaction relationship prediction of DoSWEETs

The protein sequences of DoSWEETs were submitted to STRING (https://cn.string-db.org/, accessed on May 5, 2022) [66] to identify protein-protein functional interactions. All identified interaction partners were gathered and searched using *A. thaliana* as the reference organism with the default parameters.

### Plant materials and stress treatment

The tissue-cultured *D. officinale* plantlets were grown on Murashige and Skoog (MS) medium [67] containing 30 g·L^–1^ sucrose, 7 g·L^–1^ Agar, 0.4 mg·L^–1^ 6-benzylaminopurine (6-BA) and 0.1 mg·L^–1^ 1-naphthylacetic acid (NAA) with pH 5.8 at 24 ± 1 °C under a set photoperiod of 16-h light /8-h dark. The plants were transferred to fresh medium every 8 weeks.

After 6 weeks of culture, the plantlets with the same growth status were used for sampling or stress treatment. For tissue-specific expression determination, the roots, stems, and leaves of *D. officinale* were collected. For low temperature treatment, the *D. officinale* plantlets were subjected to 0℃ for 20 h, 25 °C was used as the control. For simulating drought stress, *D. officinale* plantlets were treated with or without 20% (w/v) PEG6000 for 24 h. For MeJA treatment, the plantlet leaves were sprayed with 0.25% ethanol solution with or without 1 mM MeJA for 4 h. The leaves of stress-treated plantlets were collected and quickly frozen in liquid nitrogen for RNA extraction and detection of gene expression. All experiments were repeated for three times.

### RNA extraction and RT-qPCR

Total RNA was isolated using the EASYspin Plus Complex Plant RNA Kit (Aidlab, Beijing, China), followed by reverse-transcription using the TRUEscript 1st Strand cDNA Synthesis Kit (Onestep gDNA Removal). RNA quantity was evaluated by NanoDrop 2000 (Thermo Fisher Scientific, Wilmington, DE, USA). RT-qPCR was performed using the 2× Sybr Green qPCR Mix (Low ROX) (Aidlab, Beijing, China) with QuantStudio 3 (ABI, California, USA) in a total reaction volume of 20 µL. The PCR cycles were as follows: 95 °C for 2 min, followed by 40 cycles of 95 C for 15 s and 60 °C for 30 s. *DoGAPDH* was used as an internal control gene [68]. The relative expression levels of *DoSWEETs* were calculated using the 2^−ΔΔCt^ method [69]. ΔΔC_t_ = (C_t, Target gene_ − C_t, DoGAPDH_) − (C_t, Target gene_ − C_t, DoGAPDH_)_Max_. Fold changes of *DoSWEETs* under stresses were calculated as 2^−ΔΔCt^_Treatment_/2^−ΔΔCt^_Control_ and the values of log_2_ fold change were used to draw Fig. 5c. All experiments included three independent biological replicates. The primers used in this study are listed in Additional file 8.

### Statistical analysis

Visualization of RT-qPCR was performed using GraphPad Prism 9 (accessed on March 22, 2022). The tissue-specific expression data of *DoSWEETs* (Fig. 4d) were analyzed by Duncan’s multiple range test using one-way ANOVA program of SPSS 25 (*P* < 0.05) (accessed on March 22, 2022). Significance analysis of *DoSWEETs* expression under different stresses compared to the control (Fig. 5c) was determined by two-tailed Student’s *t*-test in SPSS 25.

## Electronic supplementary material

Below is the link to the electronic supplementary material.

Supplementary Material 1

Supplementary Material 2

Supplementary Material 3

Supplementary Material 4

Supplementary Material 5

Supplementary Material 6

Supplementary Material 7

Supplementary Material 8

## Data Availability

The datasets generated and/or analyzed during the current study are available in the NCBI Genome database with accession number GCA_019514585.1 [9] and SRA database with accession number PRJNA314400 [32], PRJNA432825 [33], PRJNA561268 [34] and PRJNA732289 [35]. The sequence alignment file for phylogenetic tree construction is available in Zenodo (10.5281/zenodo.7947133).

## Abbreviations

MeJA: Methyl jasmonate
SWEET: Sugars will eventually be exported transporters
TM: Transmembrane domain
