# Supplementary material for: Genome-wide identification, characterization and transcriptional profile of the SWEET gene family in Dendrobium officinale
Source: BMC Genomics. 2023 Jul 6;24:378. doi: 10.1186/s12864-023-09419-w (PMC10324200; doi:10.1186/s12864-023-09419-w)
Supplement: Supplementary file 2 — Supplementary Material 2 [file 12864_2023_9419_MOESM2_ESM.docx]

**Supplementary Figures**

**Supplementary Figure 1**. The phylogenetic tree with actual branch lengths and scale constructed by MEGA 11. Branch lengths shorter than 0.1 are hidden. The scale bar represents 0.5 amino acid substitution per site. The numbers above the branches indicate the bootstrap values (%) for 1000 replications. Clade I, II, III, and IV are marked by green, gray, blue, and purple, respectively.


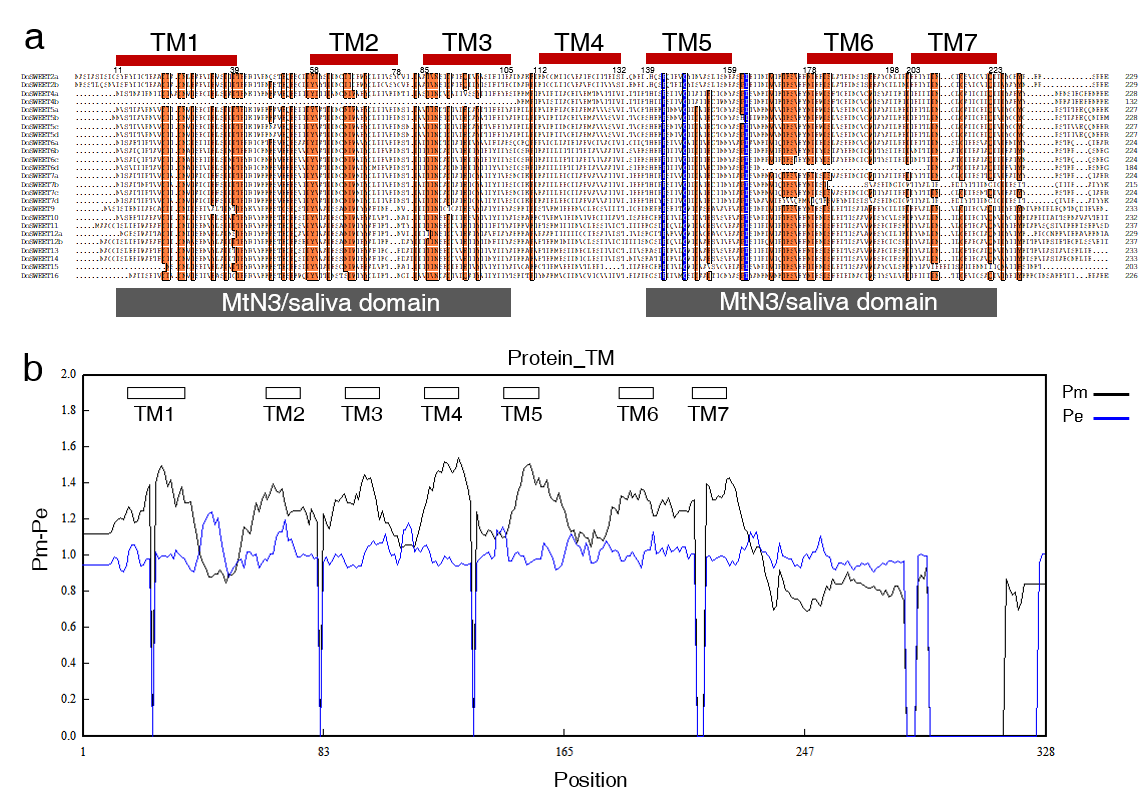


**Supplementary Figure 2. Conserved TMs analysis of DoSWEETs using DNAMAN 7.0. a** Multiple alignment of DoSWEETs. The regions highlighted with orange indicated homology > 75%, the regions highlighted with yellow indicated homology > 50%. **b** TM plots of DoSWEETs. The regions with Pm values greater than 1 and larger than that of other fragments were with greater probability of transmembrane. TM, transmembrane domain; Pm, propensity of the middle; Pe, propensity of the end.


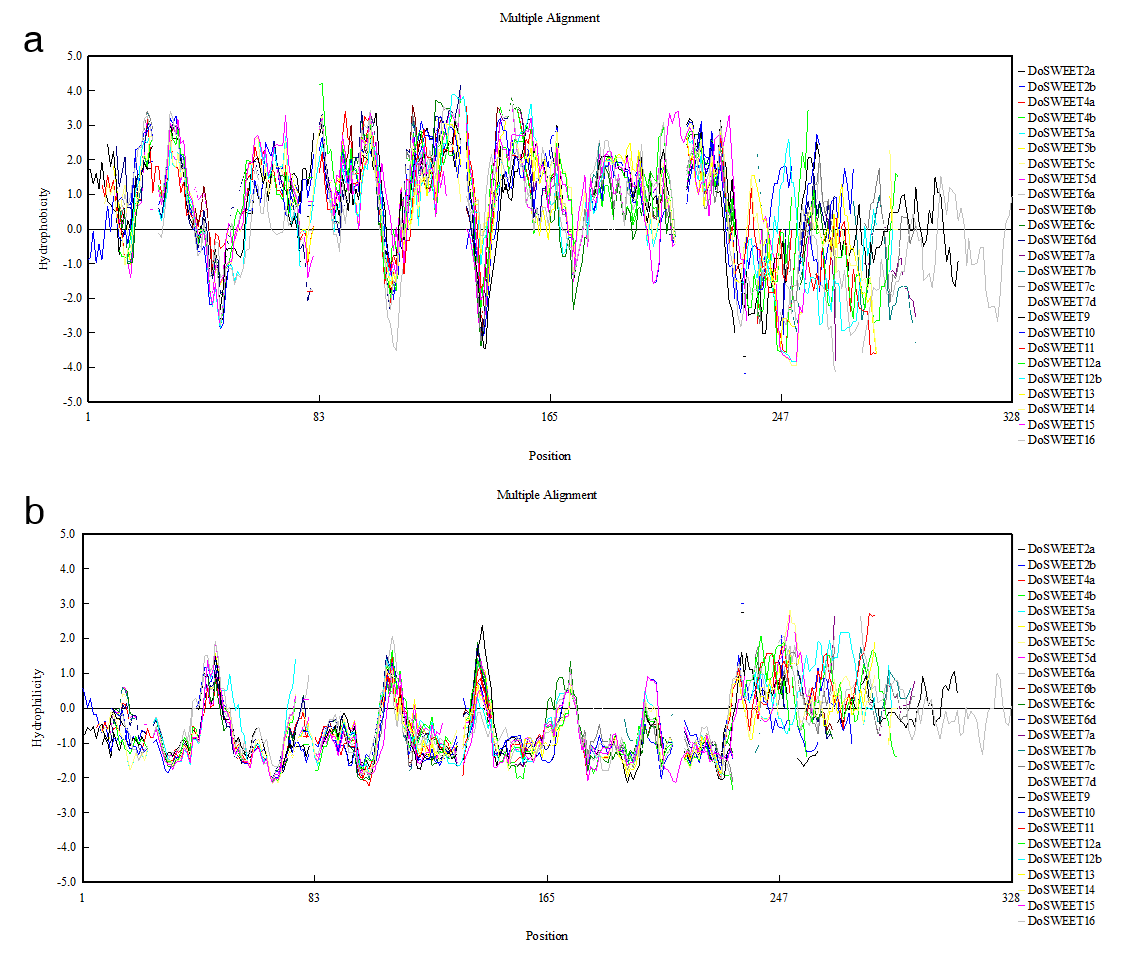


**Supplementary Figure 3. Hydrophobicity and hydrophilicity analysis of DoSWEETs using DNAMAN 7.0.** **a** Hydrophobicity value of DoSWEETs. Value > 0 indicated the hydrophobicity and regions with greater value were more hydrophobic. **b** Hydrophilicity value of DoSWEETs. Value > 0 indicated the hydrophilicity and regions with greater value were more hydrophilic.


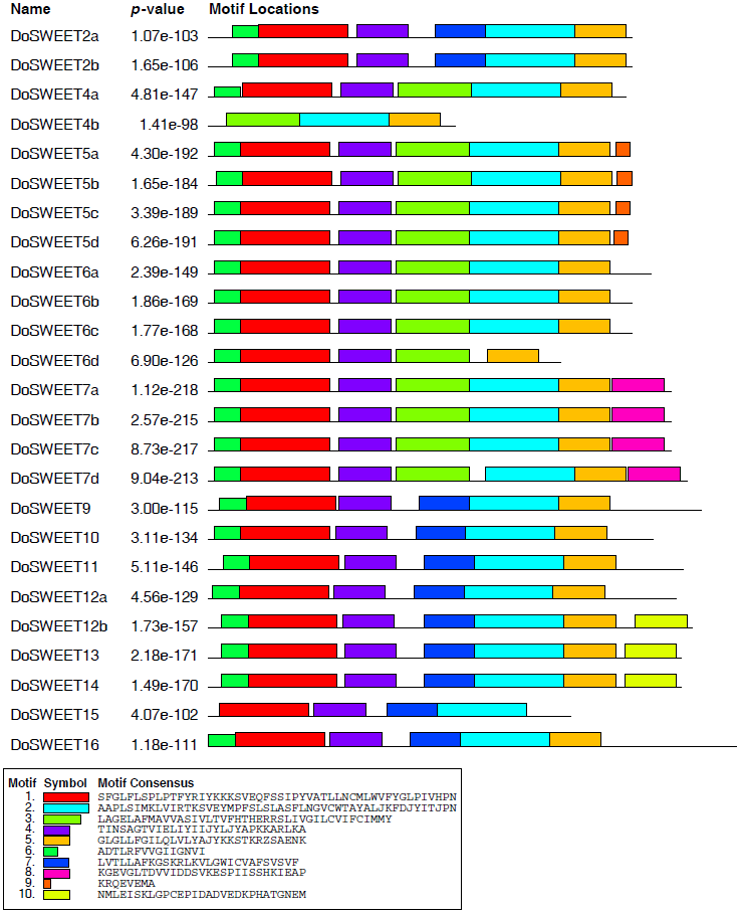


**Supplementary Figure 4. Conserved motif analysis of DoSWEET proteins by MEME.** Ten conserved motifs were predicted in DoSWEETs. The motifs were marked in different colors and the sequences were shown in the frame bellow.
